# Pasteur's legacy persists: an interview with Pascale Cossart

**DOI:** 10.1242/dmm.050009

**Published:** 2022-12-12

**Authors:** Pascale Cossart

**Affiliations:** The Institut Pasteur, 25-28 Rue du Dr Roux, 75015 Paris, France


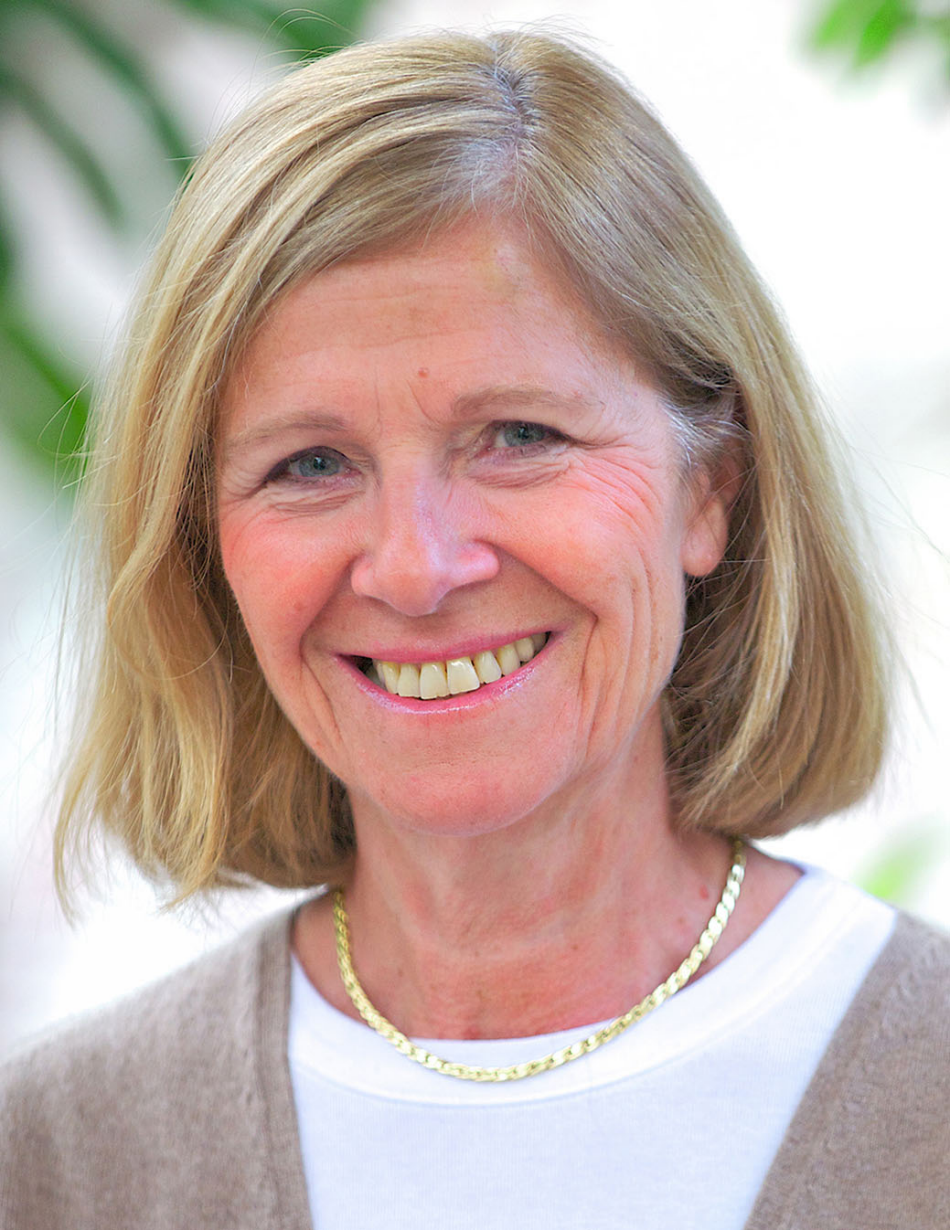


Professor Pascale Cossart ushered in the era of cellular microbiology, making seminal findings that have dramatically changed our understanding of infectious disease. She is an authoritative figure on *Listeria monocytogenes* – the common food-borne pathogen that can cause encephalitis, meningitis and miscarriage, among other conditions – making it one of the most well-studied intracellular pathogens. She unveiled how this bacterium enters and moves within and between cells and tissues while evading host defences, with many of these findings translating across bacterial species, and impacting clinical approaches to prevent and treat infectious disease. Her research has progressed and adapted alongside developments in several disciplines, with recent work exploring how bacteria induce epigenetic changes in the host during infection.

Much of Pascale's career has been within the Institut Pasteur in Paris, where she held leadership roles, including ‘Professeur de Classe Exceptionnelle’ and was the Head of the Bacteria-Cell Interactions Unit. She has been the recipient of many awards, including L'Oreal-UNESCO Award for Women in Science in 1998, as well as being appointed the Perpetual Secretary of the French Academy of Sciences in 2016. Among these accolades, she was awarded the Louis Pasteur Gold Medal in 2000, which is given every ten years by the Swedish Society of Medicine to a scientist who has excelled in the field of bacteriology. In this interview she reflects on Louis Pasteur's legacy in the field of microbiology, as we mark 200 years since his birth, and how her own work has evolved as this field continues to advance further.

**As you know, 2022 marks the 200th anniversary of the birth of Louis Pasteur. In your opinion, what have been the biggest advances in infection biology in the past 200 years and how have they shaped your research?**

Initially, Louis Pasteur and Robert Koch, and lots of other people were mostly working to determine which pathogen induced which infectious disease; and then there was a long period when people were cataloguing pathogens and disease. But I think the big, big advance was when we started to merge molecular biology and cell biology to start cellular microbiology. Cell biology was used in virology before bacteriology but virologists were not generally investigating the cellular aspects in detail. They were mostly investigating how a virus replicates. I think that, in the late eighties to early nineties, cellular microbiology was a big revolution. The next big step occurred when genomics arrived; the whole field totally changed. We were not seeing just the microbe; we were seeing its genome.

“Although I had no background in cell biology, for me, it was obvious that we would have to incorporate it into our research. I think this has shaped my research, and I also think my research and that of other scientists using similar approaches have shaped the field.”

And you also asked me how these things have shaped my research. I think I arrived at a good moment, and I participated in pushing these things forward. I could see that we were at transition phase. Although I had no background in cell biology, for me, it was obvious that we would have to incorporate it into our research. I think this has shaped my research, and I also think my research and that of other scientists using similar approaches have shaped the field.

I had been sequencing DNA before starting to work on *Listeria*. I sequenced the first gene at the Institut Pasteur, *thrA* [that encodes the *Escherichia coli* enzyme bifunctional aspartoaspartate kinase I/homoserine dehydrogenase 1] (Katinka et al., 1980), and I had sequenced the *E. coli crp* gene [that encodes the transcription activator, cAMP-activated global transcriptional regulator CRP (Crp) (Cossart and Gicquel-Sanzey, 1982), so I knew how to sequence. So, when sequencing technology became less expensive and more efficient, of course, I was ready. I remember going to the big *Listeria* meeting called the ‘International Symposium on Problems of Listeriosis’, in Denmark I think, and I said, ‘Dear colleagues, we should all work on the same strain because, soon, we will sequence the genome of *Listeria*’. At that meeting, I had brought samples of the strain on which we were working to give to everybody – and I came back with all my samples! Nobody was interested because, I think, they did not believe me. After we obtained a significant budget, it still took us two years – and we were six groups – to sequence the genome of *Listeria monocytogenes* (Glaser et al., 2001).

**Why did you choose to study *Listeria* in particular?**

Before working with *Listeria*, I was working on DNA–protein interactions; I was just working with one other scientist, Brigitte Gicquel-Sanzey, in the Institut Pasteur. We wanted to extend our team but the Institut Pasteur authorities thought that working on DNA–protein interactions was too competitive and that researchers in the United States were already better organised to do that. They told us we should work on infectious disease because we were in the Institut Pasteur. I was disappointed, but Brigitte less so, as she had been working with viruses previously. So, we decided to visit several labs in the Pasteur, to get a sense for where there was a need for investigation. It was very, very interesting and we had the time to take our time: we had time because we had permanent positions and there was no urgency to publish! So, we really interrogated many people and realised that this was the beginning of a period where people were starting to focus their own research on specific organisms. For instance, at that time Stanley Falkow was focusing on both *Salmonella and Yersinia*. It was also the period of the re-emerging tuberculosis epidemic that followed the HIV epidemic. Quickly, we started to realise that intracellular pathogens are important public health problems and it was, thus, important to understand how they were becoming intracellular and staying intracellular. By discussing this goal with several colleagues, we concluded that we should work on *Mycobacteria* – but these bacteria were known to be difficult to work with. In addition, after discussing with Patrick Berche, we decided that *Listeria* might be a good bacterium option to work with, as it is an intracellular pathogen, was less important as a public health problem but appeared to be a good model for infection. *Listeria* attracted our interest because it causes a foodborne disease that is not restricted to the intestine and can also lead to meningitis or abortion or, at least, infection of the foetus. Moreover, the genus *Listeria* was known to be comprised of both pathogenic and non-pathogenic species. I was already figuring out that comparing pathogenic and non-pathogenic species would be interesting. Also, it was known that the *Listeria* grows quickly and can easily infect tissue and cells in the lab. So, altogether, *Listeria* appeared very intriguing.

So, Brigitte and I – two people – had decided to work with two models. One [*Mycobacterium*], we knew, was responsible for an important disease. The other [*Listeria*], we knew, was an interesting model. In addition, the group of Patrick Berche isolated a non-hemolytic mutant of *Listeria* and suggested to collaborate.

Working on both *Mycobacteria* and *Listeria* was not a decision made in one hour and around a table, it was something that Brigitte and I thought about for six months. And then we applied for grants. After six months of both of us working on both bacteria, we decided that it would be better if we each choose a pathogen according to our feelings. And that's how I ended up working on *Listeria* for many, many years.

**What discoveries have you found most exciting during your career?**

Many people think that I've only worked with *Listeria* but I worked on other things before. With Richard Ebright and John Beckwith, I worked on *crp* mutants in *E. coli* that altered the specificity of Crp protein binding to DNA (Ebright et al., 1984), which was very interesting.

“So, I think there are many things that *Listeria* have taught us. It is true that when I put together all the discoveries that we made with my colleagues, I am really proud. I had fantastic team members!”

Then when I was working with *Listeria*, I loved the story of the discovery of internalin and its host receptor, E-cadherin [that enable entry of *Listeria* into non-phagocytosing cells] (Gaillard et al., 1991), which was very exciting. I was also very proud when we generated a transgenic mouse that expresses human E-cadherin to make the mice sensitive to *Listeria* infection, which had previously been a big issue (Lecuit et al., 2001).

Of course, the discovery of the *actA* gene allowing actin-based movement of *Listeria* was just, “Wow!” (Kocks et al., 1992). I will never forget that. And then I think the RNA thermosensor PrfA was a really nice discovery. We found that pathogens, such as *Listeria*, do not express certain virulence factors when they are in wild environments but then, suddenly, they express different types of virulence factor when PrfA is activated at 37°C (Johansson et al., 2002).

So, I think there are many things that *Listeria* have taught us. It is true that, when I put together all the discoveries that we made with my colleagues, I am really proud. I had fantastic team members!

**You originally studied chemistry but migrated towards cellular microbiology, similar in some ways to Louis Pasteur. What inspired this change?**

I was a chemist because I liked chemistry. I liked it at school and went on to study it at university and started to work in a chemistry lab. But then I went to an inaugural lecture of biochemistry and I thought, ‘Wow, this is making sense of all of the things that I have studied, and this is what I really like’. So, I went to see the professor and I changed lab, and I started to do biochemistry. Then I did my PhD at the Institut Pasteur on sequencing proteins, which was really chemistry of proteins. Then DNA-sequencing technology arrived, so I switched from protein chemistry to studying DNA. And from DNA I started to be interested in bacteria and genetics. Then when I started to work with *Listeria*, I had to switch to cell biology. I have always been switching disciplines. Since I had no formal background in biology, when I wanted to study anything, I had to go to biology meetings to learn.

**To celebrate this bicentenary, the Institut Pasteur has been organising many events, some of which you have been involved in. You dedicate a lot of your time to organising courses and events – why is this endeavour important to you?**

I am organising or coordinating a lot of events around the bicentenary because I think it's important that people realise that microbiology is more than ever a very important field for human beings. I think people have forgotten or ignore what microbes can do. With the discovery of antibiotics people thought infectious disease would disappear. But suddenly, even more with the pandemic, people realise that microbes are very important. But people are also realising that microbes may not always  cause disease; they may also be important for the health of humans, animals and the environment. Yes, we are in a period where more people are realising that microbes are interesting. And what is very striking is that immunologists are now really studying microbes, because for a long time, they were only working with chemicals. Also, neurobiologists are becoming interested in microbiology. All biologists are becoming interested in microbes!

“Microbes are invading all fields of biology.”

Microbes are invading all fields of biology. I think it's fantastic for microbiologists. I must say that, when I started, microbiology was not considered very interesting. Even in the Institut Pasteur, people who had won the Nobel Prize for their work with microbes switched to the study of eukaryotes, working on mice, *Drosophila* and other model systems. So, we were very few to still work with bacteria. But then, when we switched to studying pathogens, there was a new dynamic in the Institut Pasteur. And now there is another new dynamic, which is that microbes are everywhere.

**Looking beyond the bicentenary of Pasteur's birth – what's next for infection biology?**

So, what is next? I think that when people are studying infection biology, they have to do *in vivo* studies and they have to ask themselves whether they are studying their favourite microbe infection in the correct way. Are you using the right tissue or cell type, for instance? I think we have to also consider the time of the day we are studying an infection and we have to become more conscious of which other organisms are present when one is studying a specific microbe. And, within the microbiota, what is the role of each of the microorganisms present? So, it's becoming more complex but I think that's where the infection biology field has to go.

**You held several leadership roles throughout your career. How important is it for young female researchers to see women in these roles?**

Frankly, I think if you want to be taken seriously, you should accept these roles if they are offered. At one stage of your career, you have to take roles like the head of a department or institute, otherwise you are always assisting other people. That's what I did and I think I did it correctly. Of course, it takes your time but you cannot always be fed – you have to feed other people. I think it's important to show people that it's doable and that doing this does not prevent you from being successful in your own research.

**You have also been the recipient of many awards, acknowledging not only your research but also your role as a mentor. What strategies have you adopted to become a great mentor?**

Well, I don't think I had a strategy. When I was 20, I did not have a big plan to get to a certain place for when I would be 60. You just have to adapt to the situation and evolve. To tell you the truth, I had never thought that I would be where I am. I just wanted to enjoy myself and to do research. In fact, when I started, people were not talking about career and future, and all that. We were just enjoying life. We were lucky to have  a permanent position very early on and to be able to do research. Then of course, it changed. I never had to, for example, apply to as many positions as people have to now.

In terms of mentoring advice, I think you need to be very positive. I am a positive person! It is important to share your positive attitude and enthusiasm with your colleagues. I am not a critical person either. If I go to a seminar, in most cases, I'm happy and take something out of it. I can be critical and think that someone should have less busy slides, for instance, but I always try to take something out of it.

I have also tried to spend time with people in my lab. I have always been amazed when I see labs where there are 12 students. I've had maybe a maximum of four students but a lot of postdocs. It is important for a student to be very well mentored.

I also advised my people to go with your feelings. If you don't like your research anymore and you don't find it interesting then, if possible, change subject. Always do things that you like.

**You are very passionate about science but were there any alternative career paths you considered pursuing?**

I know that I am passionate about science, and I don't force myself to be passionate. Why? Because I am interested in how things work. And I am interested in the details of a mechanism because I am a chemist. If you look at my main research discoveries, I always went to the nucleotide or amino acid level.

But I am passionate about other things. For example, I like gardening and cooking. For me, cooking is like chemistry. I like to have friends at home and to cook for them. I don't buy things that are already cooked.

Did I consider another job at any time? No, never. I always thought that I was happy where I was. I don't know if I would have been able to do another job. Except, maybe, run a big department or Institute. I knew I could do that and that's why I accepted to be the Perpetual Secretary of the French Academy of Sciences. I knew I could think about what needed to be done and be organised.
